# Functional Capacity Evaluation in Different Societal Contexts: Results of a Multicountry Study

**DOI:** 10.1007/s10926-018-9782-x

**Published:** 2018-05-25

**Authors:** Jone Ansuategui Echeita, Matthias Bethge, Berry J. van Holland, Douglas P. Gross, Jan Kool, Peter Oesch, Maurizio A. Trippolini, Elizabeth Chapman, Andy S. K. Cheng, Robert Sellars, Megan Spavins, Marco Streibelt, Peter van der Wurff, Michiel F. Reneman

**Affiliations:** 1 Department of Rehabilitation Medicine, University Medical Center Groningen, University of Groningen, P.O. Box 30.002, 9750 RA Haren, Groningen, The Netherlands; 2 Institute of Social Medicine and Epidemiology, University of Lübeck, Lübeck, Germany; 3 Institute for Sports Studies, Hanze University of Applied Sciences, Groningen, The Netherlands; 4 Department of Physical Therapy, University of Alberta, Edmonton, AB Canada; 5 Rehabilitation Centre Valens, Valens, Switzerland; 6 Center for Disability Research, Liberty Mutual Research Institute for Safety, Hopkinton, Boston, USA; 7 PhD in Rehabilitation Sciences Program, Institute for Health Professions, Massachusetts General Hospital (MGH), Charlestown, Boston, USA; 8 Department of Work Rehabilitation, Rehaklinik Bellikon, Suva Care, Bellikon, Switzerland; 9 Toyota Motor Manufacturing Canada, Puslinch, ON Canada; 10 Ergonomics and Human Performance Laboratory, Department of Rehabilitation Sciences, The Hong Kong Polytechnic University, Hong Kong, China; 11 FCE Systems Ltd, Geraldine, New Zealand; 12 Occupational Therapy Inc., Randburg, South Africa; 13 Department of Rehabilitation, German Federal Pension Insurance, Berlin, Germany; 14 Research & Development, Military Rehabilitation Center Aardenburg, Doorn, The Netherlands; 15 Institute for Human Movement Studies, HU University of Applied Sciences, Utrecht, The Netherlands

**Keywords:** Internationality, Occupational health, Sociological factors, Lifting, Chronic pain

## Abstract

*Purpose* To examine factors associated with Functional Capacity Evaluation (FCE) results in patients with painful musculoskeletal conditions, with focus on social factors across multiple countries. *Methods* International cross-sectional study was performed within care as usual. Simple and multiple multilevel linear regression analyses which considered measurement’s dependency within clinicians and country were conducted: FCE characteristics and biopsychosocial variables from patients and clinicians as independent variables; and FCE results (floor-to-waist lift, six-minute walk, and handgrip strength) as dependent variables. *Results* Data were collected for 372 patients, 54 clinicians, 18 facilities and 8 countries. Patients’ height and reported pain intensity were consistently associated with every FCE result. Patients’ sex, height, reported pain intensity, effort during FCE, social isolation, and disability, clinician’s observed physical effort, and whether FCE test was prematurely ended were associated with lift. Patient’s height, Body Mass Index, post-test heart-rate, reported pain intensity and effort during FCE, days off work, and whether FCE test was prematurely ended were associated with walk. Patient’s age, sex, height, affected body area, reported pain intensity and catastrophizing, and physical work demands were associated with handgrip. Final regression models explained 38‒65% of total variance. Clinician and country random effects composed 1–39% of total residual variance in these models. *Conclusion* Biopsychosocial factors were associated with every FCE result across multiple countries; specifically, patients’ height, reported pain intensity, clinician, and measurement country. Social factors, which had been under-researched, were consistently associated with FCE performances. Patients’ FCE results should be considered from a biopsychosocial perspective, including different social contexts.

**Electronic supplementary material:**

The online version of this article (10.1007/s10926-018-9782-x) contains supplementary material, which is available to authorized users.

## Introduction

The use of functional capacity evaluations (FCEs) has become part of common clinical practice in several areas of occupational and rehabilitation medicine on patients with musculoskeletal diseases [1–3]. FCEs have been defined as: “An evaluation of capacity of activities that is used to make recommendations for participation in work while considering the person’s body functions and structures, environmental factors, personal factors and health status” [4]. Due to the influence these tests have on return-to-work, disability claims, compensation, and treatment planning decisions [5], a considerable amount of research has been conducted to determine the psychometric properties of various FCE protocols, especially construct validity and what factors influence FCE results [6–8].

Multiple factors including personal, health care, professional, legal, administrative, and cultural characteristics have been shown to influence work participation [9]. Due to the significant associations found between FCE results and physical, psychological, and social factors in healthy workers and in different patient groups [4, 10], a similar biopsychosocial explanatory model could be applied to FCE. However, to date, the majority of research on FCE has focused on physical and psychological factors, among which are sex, age, functional self-efficacy, and patients’ reported pain and disability [11–13].

Within the International Classification of Functioning disability and health (ICF) framework, social factors have been defined as “Factors that make up the physical, social and attitudinal environment in which people live and conduct their lives. These factors are external to individuals and can have a positive or negative influence on the individual’s performance as a member of society, on the individual’s capacity to execute actions or tasks, or on the individual’s body function or structure” [14]. Only a few studies have highlighted the relevance of social factors on FCE results [15]. International studies have shown differences in FCE results between individuals from different cultural groups within a country [16, 17] and between countries [18]. Furthermore, some studies suggest that functional capacity depends on the clinician’s level of fear-avoidance behavior and instructional strategy, as demonstrated in samples of healthy subjects and of patients, respectively [19, 20]. The evidence of these studies, although limited, suggests that FCE results vary between societal contexts. Additionally, the majority of the research on determinants of FCE performance has been performed on relatively small patient samples (n < 100 patients), making large multiple regression prediction models unstable. These studies have also come from a limited number of countries. Considering that countries differ in their insurance, compensation, and health system characteristics, the generalizability of such studies beyond those countries’ individual characteristics may be limited.

The present study was designed to further examine the determinants of FCE performance in patients from different societal contexts. The obtained knowledge may help better explain the underlying differences in FCE results and, in turn, allow better interpretation. More knowledge of a patient’s biopsychosocial context, including personal, health care, professional, legal, administrative, and cultural characteristics, and how it influences performance may lead to more effective and efficient delivery and interpretation of FCE services. Therefore, the aim of the current study was to examine what biopsychosocial factors are associated with FCE results in patients with painful musculoskeletal conditions, with a focus on social factors across multiple countries or societal contexts.

## Methods

### Design

A cross-sectional, observational study was performed within care as usual between September 2015 and April 2016. Ten participating countries joined the study: The Netherlands, Canada, Switzerland, Germany, Austria, South Africa, Australia, New Zealand, China, and the United States of America.

### Procedure

Initially, the project team developed a study protocol. Next, participants for the study were recruited through snowball sampling. Contacts involved in FCE research from different countries were approached and asked to join the study as representatives of their countries. In addition to being in charge of the development of the study in their country and data collection as per protocol standards, the representatives were responsible for recruiting clinicians involved in FCE assessments from various facilities within their country. Participating clinicians assisted in the enrollment of patients undergoing FCE assessments. Informed consent was obtained from participating patients and clinicians. Participating clinicians were asked to complete a set of questionnaires prior to patient’s study enrollment. Participating patients were asked to complete additional questionnaires before performing FCE tests. Data from three FCE domains were collected: material handling (floor-to-waist lift), energetic capacity (six-minute walk test), and hand and finger strength (handgrip strength).

Approval to perform the study was obtained from the relevant research ethics board of the countries where data were collected. All procedures were in accordance with the ethical standards of the Helsinki Declaration of 1975 as revised in 2014 [21].

### Participants and Societal Context

#### Patients

Patients who were to be tested with FCE and met the inclusion criteria were eligible to participate in the study. Inclusion criteria for patients were: adult patients over 18 years of age with non-specific sub-acute or chronic musculoskeletal pain, and with sufficient language skills to understand the instructions. Excluded were patients who were pregnant, retired, on permanent sick-leave, who had specific musculoskeletal diagnoses (i.e. fractures, tumors, radicular syndromes), or who had co-morbidities affecting performance or safety during FCE (i.e. cardiovascular conditions).

#### Clinicians

Because clinicians’ characteristics were considered a potentially relevant social variable that could influence FCE results, clinician data were collected. Clinicians who administered FCE in routine clinical practice and met the inclusion criteria were eligible to participate. Inclusion criteria for clinicians were: FCE-trained clinicians with at least 1 year of experience conducting FCE, more than 20 FCEs administered, and sufficient understanding of English to complete the compulsory questionnaires.

#### Societal Context

Participants were recruited from one or more facilities from each country. System or societal characteristics of the eight countries that participated in the study were collected and are presented in Online Resource 1.

The total sample was composed of patients from different contexts. Dutch sample was obtained from two outpatient rehabilitation centers referred by their company doctor for a multidisciplinary assessment. Canadian sample was obtained from one rehabilitation center, where individuals undergoing FCE were injured during motor vehicle accidents or were off work due to non-work related injuries. Patients were tested to determine disability related to previous employment (return-to-work) or activities of daily living (ADL), depending on whether the person was working prior to the injury. Swiss sample was obtained from three inpatient and outpatient rehabilitation centers. Inpatients were referred for a 3-week rehabilitation program, and underwent FCE for therapy planning and return-to-work assessment; whereas outpatients were referred by their disability insurance company to determine level of work related disability. German sample was recruited from six multi-professional work-related medical rehabilitation settings. These patients had musculoskeletal disorders and were performing FCE prior to admission to a 3-week rehabilitation program. Austrian sample was obtained from one rehabilitation center run by the AUVA (General Accident Insurance Institute). Patients were manual workers who had experienced an accident at work and were performing FCE for return-to-work assessment. South African sample was obtained from three facilities. One facility performed FCEs to determine work-related disability for insurance companies; whereas the others were a medico-legal practices where the FCE was done to assist in case settlements after road accidents. New Zealander sample was comprised of long-term claimants who had not returned to work after failure to respond to rehabilitation. Patients underwent FCEs for return-to-work assessments, therapy continuation assessments, or to determine disability. Chinese sample was obtained from one hospital in Hong Kong. Patients performed the FCE for return-to-work assessments and prior to participating in work hardening rehabilitation programs.

### Measurements

#### FCE Variables

This study allowed for a variety of FCE protocols as long as they had demonstrated reliability on the lifting test in peer-reviewed articles. The compulsory FCE measurements included are discussed in detail below.

##### Floor-to-Waist Lift Test

The test characteristics described below are in accordance with the WorkWell protocol (former Isernhagen Work Systems) [6], the WEST-EPIC protocol [22] or the Blankenship protocol [23, 24]. These protocols differ in the type of material and the standardization of the instruction. The WorkWell protocol was operated as a progressive performance test, which began with an easily lifted weight that was gradually increased until the evaluator determined a “safe maximum lift” or until the patient stopped lifting. Patients were instructed to perform repetitive lifting series of a loaded box with as much weight as safely possible from a shelf at waist height to the floor, and back to the shelf. From the initial weight until the “safe maximum lift” weight, five lift repetitions were made with each weight. The safe lifting endpoint has been defined as the maximum load a patient could lift five times, while maintaining a stable spine and without exceeding the patient’s physiological limits [i.e. heart-rate (HR)]. The WEST-EPIC protocol was conducted as a progressive performance testing. The lift test was divided in cycles, which were composed of three subtests (knuckle-to-shoulder, floor-to-knuckle and floor-to-shoulder). These cycles were performed at two frequencies each before incrementing the weight, one lift per subtest and, if the patient was capable, four times. The lift test began with an empty standardized crate of 4.5 kg, which was gradually loaded with masked weights. Patients were blind to any load during the test. After each cycle they were asked whether they would be able to perform that task on a “safe and dependable manner eight to twelve times a day”. The “maximum acceptable load” was identified by observing the patient’s HR, posture and body mechanics, and psychophysical response. The Blankenship protocol was performed as a progressive lifting test. The lift test was used to determine how much weight the patient was able to lift at an occasional frequency (0–33% of the workday). The lift test began with an empty standardized crate of 4 kg, which was gradually loaded with weights to a maximum weight decided by the patient. Aspects of reliability and validity have been studied for all FCE protocols performed in this study [22, 23, 25, 26].

Clinicians recorded patient’s maximum weight lifted in kilograms along with HR before and after the test, patient-reported effort measured with Borg’s CR-10 scale [27], and clinician’s observed physical effort [28, 29]. In addition, the reason for ending the test was recorded [30].

##### Six-Minute Walk Test (6MWT)

The 6MWT was performed according to the recommendations of the American Thoracic Society [31]. The test was carried out on a flat hard surface, where two markers (i.e. tape, traffic cones) were set 30 m apart. Patients were instructed to walk back and forth between the two markers as much as possible at their own pace for 6 min. Running or jogging was not allowed; however, patients were able to stop and rest during the test. The 6MWT has shown acceptable test–retest and inter-rater reliability, criterion validity and acceptability in adults with chronic pain, fibromyalgia and chronic fatigue [32]. In addition to the distance walked in meters, patient’s HR before and after the test, patient-reported effort measured with Borg’s CR-10 scale [27], whether the test was prematurely stopped, and the reason for ending the test [30] was recorded.

##### Handgrip Strength Test

Grip strength measurements were taken with an adjustable-handle dynamometer. For standardization, Jamar dynamometer (or compatible device) was set in the second handle position. Following the procedure described by Mathiowetz et al. [33], patients were seated with their shoulder adducted and neutrally rotated, elbow flexed at 90°, forearm in neutral position, and wrist between 0° and 30° dorsiflexion and between 0° and 15° ulnar deviation. In that position, they were instructed to squeeze the dynamometer as hard as possible for three successive trials, left and right hand separately. The mean grip-strength of each hand was calculated and recorded in kilograms. The handgrip strength test has demonstrated acceptable reliability in healthy patients and patients with cervical radiculopathy [34].

#### Biopsychosocial Variables

Data from healthcare, workplace, legislative, and personal systems as well as clinician and patient characteristics were collected [35].

##### Patients’ Demographic Characteristics

Age; sex; height, weight, body mass index (BMI); affected body area, duration of pain; country whose social system applied to the patient; cultural background as measured by nationality; mother language; educational level; employment characteristics: job and physical work demands per Dictionary of Occupational Titles (DOT); work status; days off work due to pain; and compensation status.

##### Brief Psychological Screening

Eight self-reported screening questions for five psychosocial risk factors associated with pain [36]: depression, anxiety, social isolation, catastrophizing, and fear of movement. The response options were standardized in a 0 to 10 scale, where lower scores indicated lower risk. Moderate to high correlations with full-length questionnaires have been demonstrated for anxiety, depression, social isolation, catastrophizing, and fear of movement [36].

##### Disability (Pain Disability Index—PDI)

A 7-item self-reported questionnaire measuring the degree to which pain interferes with functioning across a range of activities: family/home responsibilities, recreation, social activity, occupation, sexual behavior, self-care, and life-support activity. The score for each item ranges from 0 (no interference) to 10 (total interference) and the total score can range from 0 to 70, where 70 indicates a total interference on life activities. The PDI has been shown to be a valid and reliable measure of pain-related disability, and shows sufficient internal consistency [37].

##### Pain Intensity (Numeric Rating Scale—NRS)

A self-reported scale to measure the current pain intensity in adults. The scale ranges from 0 (no pain) to 10 (worst possible pain). The reliability and validity of the NRS has been established for patients with rheumatic pain conditions [38].

##### Work Ability (Work Ability Score—WAS)

A single-item question of the Work Ability Index (WAI), which measures patients’ current work ability compared with their lifetime best. This item yields a score between 0 (unable to work) and 10 (work ability at its best). The WAS has been shown to be a good alternative to the full 28-item WAI [39].

##### Clinicians’ Demographic Characteristics

Age; sex; profession; workplace: facility, canton/province/state, country; clinical; and FCE experience.

##### Clinicians’ Pain Beliefs (Adapted Back Beliefs Questionnaire—BBQ)

A questionnaire measuring an individual’s beliefs about back trouble. For the purpose of this study, this questionnaire was adapted to measure clinicians’ beliefs about musculoskeletal pain, for which ‘back trouble’ was changed into ‘musculoskeletal pain’. The BBQ assesses the level of agreement for nine statements on a 5-point Likert scale (no agreement–total agreement). The total score can range from 9 to 45, where lower scores are related to more negative beliefs on pain. The original questionnaire has shown internal consistency and excellent reliability in workers in a manufacturing factory [40] as well as construct validity and test–retest reliability in the general population [41, 42].

##### FCE Characteristics

Purpose for undergoing FCE, whether results had a direct effect on the patient’s financial situation, type of protocol performed.

### Data Analysis

Data records from all the participating countries were merged into a single database. Some variables were recoded for statistical purposes due to uneven variable distributions (pain duration and days off work were transformed into six categories and amount of compensation into five), and to form groups of similar characteristics (work status and affected body part were converted into new variables of six and five categories each).

The dataset was checked for missing data and outliers. If more than 5% of the cases missed information, the distribution of the missing values per variable was checked by comparing the results of the FCE tests of those with missing data to those without missing data. For continuous dependent variables, *t* tests or Mann–Whitney tests for independent samples were used. The relevance of the variables with statistically significant differences was further examined by comparing the medians of the two groups with boxplots. The influence of outliers (larger than three SD) was examined with Cook’s distance and leverage values.

Descriptive statistics were calculated for patients’, clinicians’, and FCE characteristics, and presented as means and standard deviations for continuous variables, and counts and percentages for categorical variables. In order to assess the explanatory value of biopsychosocial determinants for FCE results, while considering the nested design (participants within clinicians within countries), multilevel regression analyses were performed. The models were created with patients as level 1, clinicians as level 2, and measurement countries as level 3. These multilevel models involved biopsychosocial variables as independent variables and FCE test results as dependent variables.

The multilevel modeling with MLwiN software was conducted. MLwiN uses the Restricted Iterative Generalized Least-Squares (RIGLS) method to examine a model’s goodness of fit. To establish whether the addition of a variable was a significant improvement to the model’s fit, the most recent model’s deviance (− 2 * LogLikelihood) was compared to the previous model’s. The following process was applied:

- First, 1-level and 3-level null models were built per dependent variable. To evaluate whether clinician and country had a significant effect on the dependent variables, 1-level null models were compared to 3-level null models (i.e. accounted for variance within clinicians and countries).
- Second, biopsychosocial variables were separately added as fixed effects to the 3-level null model. To determine the association of each of these with FCE test results, the 3-level null model was compared to each biopsychosocial variable’s 3-level model.
- Third, a selection of the variables to be entered into the multiple multilevel models was made. A minimum of ten measurements per variable is required for valid multiple regression models [43, 44]; therefore, the number of biopsychosocial variables included in the multiple multilevel regression models was limited. This selection of variables was made based on the statistical significance level from the simple multilevel models.
- Fourth, a series of multiple multilevel models were performed with biopsychosocial variables entered in a stepwise-forward method. When building the multiple multilevel models, only the independent variables that significantly improved the model’s fit (p < 0.05) remained. The statistical significance of the final models was established at p < 0.05.

The reported results of the simple multilevel regression analyses were fixed effect’s explained variance (R^2^) and p value. R^2^ was calculated as $(\upsigma _{0}^{2} - \upsigma _{1}^{2})/(\upsigma _{0}^{2}),$ where $\upsigma _{0}^{2}$is the variance of the 3-level null model and $\upsigma _{1}^{2}$the variance of the 3-level model with the variable [45]. The reported results of the multiple multilevel regression analyses were fixed effect’s unstandardized coefficient and its standard error. Total variance explained by fixed effects was reported as a measure of the relevance of all fixed effects in the final models. To determine the total residual variance which was due to clinicians and countries, or, in other words, the correlation of the outcomes within clinicians and countries, the intraclass correlation (ICC) was calculated.

Diagnostic and descriptive analyses were performed using SPSS software version 22.0 (IBM Corp., NY) and multilevel regression analyses with MLwiN software version 2.35 (Centre for Multilevel Modelling, University of Bristol, UK).

## Results

### Characteristics of the Sample and Context

A total of 376 patients, 54 clinicians, 18 facilities and 8 different countries participated in the study. Of this sample, it was reported that three patients did not sign the informed consent form and one did not have any FCE measurement. These patients were excluded. Of the 372 patients, 261 had information on all 4 FCE measurements and 363 had either lift capacity and walk and/or handgrip strengths capacity results. In total 341 patients and all of the clinicians filled in all self-reported questionnaire items. Full datasets were therefore collected for 242 patients (64.4%).

Multicollinearity was checked before building the multiple multilevel regression models. It was found that cultural background as measured by nationality and/or mother language were also very strongly related to the measurement country in lift and handgrip strength tests. Therefore, these variables were not included in the final multiple multilevel regression models.

Patients’ biopsychosocial characteristics are shown in Table 1. Additionally, these biopsychosocial characteristics for each of the samples can be found in Online Resource 2.

**Table 1:** Biopsychosocial characteristics of the study population (n = 372); mean ± SD or n (%) are shown

	Mean ± SD or n (%)
Floor-to-waist lift performance (kg)	19.1 ± 10.4
Six-minute walk test performance (m)	479.8 ± 151.0
Right handgrip strength performance (kgF)	36.5 ± 16.9
Left handgrip strength performance (kgF)	34.8 ± 16.2
*Biological factors*	
Patient’s	
Age (years)	43.9 ± 12.5
Sex (female)	156 (41.9%)
Height (cm)	173.4 ± 9.5
Weight (kg)	82.5 ± 19.0
BMI (kg/m^2^)	27.4 ± 5.5
Affected body area	
Low back	133 (35.8%)
Lower extremity	66 (17.7%)
Upper extremity	42 (11.3%)
Neck	53 (14.2%)
Generalized	76 (20.4%)
Other^c^	2 (0.5%)
Floor-to-waist lift test	
Observed physical effort	
Light to moderate	92 (24.7%)
Heavy	106 (28.5%)
Maximum	170 (45.7%)
Test ended prematurely (yes)	124 (33.3%)
HR (bpm)	
Pre-test	86.4 ± 16.9
Post-test	116.2 ± 22.8
Six-minute walk test	
Test prematurely ended (yes)	18 (4.8%)
HR (bpm)	
Pre-test	90.7 ± 15.8
Post-test	111.4 ± 20.7
*Psychological factors*	
Patient-reported	
Pain intensity (NRS) (0–10)^a^	5 (3–7)
Pain duration	
Less than ½ year	64 (17.2%)
½–1 year	95 (25.5%)
1–2 years	69 (18.5%)
2–3 years	54 (14.5%)
3–10 years	64 (17.2%)
More than 10 years	20 (5.4%)
Effort during floor-to-waist lift test (Borg CR-10)	6.5 ± 2.2
Effort during six-minute walk test (Borg CR-10)	4.7 ± 2.5
Screening questionnaire	
Anxiety (0–10)^a^	3 (1–6)
Social isolation (0–10)^a^	2 (0–5)
Catastrophizing (0–10)^a^	4.5 (2–7)
Depression (0–10)^a^	4 (1.5–6.5)
Fear of movement(0–10)^a^	4.5 (2–7)
Disability (PDI) (0–70)^a^	30 (20–42)
Family/home responsibilities (0–10)^a^	5 (3–7)
Recreation (0–10)^a^	6 (3–8)
Social activity (0–10)^a^	5 (2–7)
Occupation (0–10)^a^	7 (5–8)
Sexual behavior (0–10)^a^	4 (1–6)
Self-care (0–10)^a^	2 (0.8–5)
Life-support activity (0–10)^a^	2 (0–5)
Work ability—single item (WAS) (0–10)^a^	4 (2–6)
*Social factors*	
Patient’s	
Living country	
The Netherlands	52 (14.0%)
Canada	5 (1.3%)
Switzerland	86 (23.1%)
Germany	100 (26.9%)
Austria	12 (3.2%)
South Africa	34 (9.1%)
New Zealand	64 (17.2%)
China	19 (5.1%)
Cultural background^b^	
Dutch	51 (13.7%)
Canadian	4 (1.1%)
Swiss	59 (15.9%)
German	100 (26.9%)
Austrian	13 (3.5%)
South African	34 (9.1%)
New Zealander	55 (14.8%)
Chinese	21 (5.6%)
Other^c^	35 (9.4%)
Mother language	
Dutch	51 (13.7%)
English	73 (19.6%)
German	171 (46.0%)
Afrikaans	9 (2.4%)
Chinese	21 (5.6%)
Other^c^	47 (12.6%)
Marital status	
Single	98 (26.3%)
Living together	7 (1.9%)
Married	197 (53.0%)
Common law	14 (3.8%)
Separated	6 (1.6%)
Divorced	42 (11.3%)
Widowed	8 (2.2%)
Educational level	
No degree	8 (2.2%)
Elementary education	35 (9.4%)
High-school education	118 (31.7%)
Vocational training	179 (48.1%)
Bachelor	26 (7.0%)
Master	5 (1.3%)
Doctorate	1 (0.3%)
Physical work demands (DOT)	
Sedentary	45 (12.1%)
Light	77 (20.7%)
Medium	117 (31.5%)
Heavy	89 (23.9%)
Very heavy	40 (10.8%)
Work status	
Working on regular duty	59 (15.9%)
Working on modified duty	32 (8.6%)
Working on reduced duty	22 (5.9%)
On full sick-leave	183 (49.2%)
Unemployed	51 (13.7%)
On disability allowance	22 (5.9%)
Other^c^	3 (0.8%)
Days off work	
No days off	64 (17.2%)
Less than ¼ year	60 (16.1%)
¼–½ year	69 (18.5%)
½–1 year	85 (22.8%)
1–2 years	47 (12.6%)
More than 2 years	42 (11.3%)
Compensated (yes)	263 (70.7%)
Amount of compensation	
Compensation at 0%	109 (29.3%)
Compensation less than 50%	9 (2.4%)
Compensation between 50 and 74%	41 (11.0%)
Compensation between 75 and 99%	148 (39.8%)
Compensation at 100%	64 (17.2%)
Clinician’s	
Age (years)	40.9 ± 15.7
Sex (female)	30 (55.6%)
Working country	
The Netherlands	4 (7.4%)
Canada	2 (3.7%)
Switzerland	22 (40.7%)
Germany	14 (25.9%)
Austria	5 (9.3%)
South Africa	4 (7.4%)
New Zealand	1 (1.9%)
China	2 (3.7%)
Profession	
Physical therapist	38 (70.4%)
Occupational therapist	12 (22.2%)
Other^c^	4 (7.4%)
Experience	
Years as professional^a^	16 (7.5–21.3)
Years as FCE assessor^a^	7 (3–11)
No FCE in the last 2 years^a^	37.5 (20–100)
Pain beliefs (adapted BBQ) (9–45)^a^	40.5 (36–43)
FCE	
Purpose	
Admission for rehabilitation	131 (35.2%)
(Pre-) employment assessment	2 (0.5%)
Return-to-work	159 (42.7%)
Case settlement	32 (8.6%)
Determine disability	47 (12.6%)
Other^c^	1 (0.3%)
Direct influence on financial status (yes)	179 (48.1%)
Type of protocol	
Work well	284 (76.3%)
WEST-EPIC	24 (6.5%)
Blankenship	64 (17.2%)
Max. HR criterion: 85% maximal HR (yes)	153 (41.1%)
Floor-to-waist lift performance	
Test ended prematurely (yes)	124 (33.3%)
Reason for ending	
Normal end of test	146 (39.2%)
Maximal allowed HR is reached	10 (2.7%)
Evaluator’s decision: safety	22 (5.9%)
Evaluator’s decision: max. capacity	83 (22.3%)
Patient’s decision	95 (25.5%)
Other: maximum time exceeded	2 (0.5%)
Six minute walk test performance	
Test prematurely ended (yes)	18 (4.8%)
Reason for ending	
Normal end of test	234 (62.9%)
Evaluator’s decision: max. capacity	21 (5.6%)
Patient’s decision	16 (4.3%)

*BMI* Body Mass Index, *HR* heart-rate, *DOT* Dictionary of Occupational Titles, *PDI* Pain Disability Index, *NRS* Numeric Rating Scale, *WAS* Work Ability Score, *FCE* Functional Capacity Evaluation, *BBQ* Back Beliefs Questionnaire

^a^Median (IQR 25–75) values are given

^b^Cultural background measured as nationality

^c^Other: patient’s affected body area: pelvic floor paralysis ex childbirth (n = 1), thoracic spine (n = 1); patient’s cultural background: Albanian (n = 1); American (n = 1), Congolese (n = 1), Croatian (n = 1), Fijian-Indian (n = 1), Indian (n = 3), Iraqi (n = 1), Italian (n = 6), Kosovar (n = 2), Liechtensteiner (n = 4), Macedonian (n = 1), Polish (n = 1), Portuguese (n = 1), Romanian (n = 1), Russian (n = 1), Serbian (n = 2), Spanish (n = 1), Tongan (n = 2), Turkish (n = 4); patient’s mother language: Albanian (n = 6), Bosnian (n = 1), Croatian (n = 2), French (n = 3), isiZulu (n = 7), Italian (n = 4), Panjabi (n = 1), Polish (n = 1), Romanian (n = 1), Sepedi (n = 1), Serbian (n = 3), SeSotho (n = 7), Swazi (n = 1), Tamil (n = 1), Tongan (n = 2), Tswana (n = 1), Turkish (n = 4), Ukrainian (n = 1); patient’s work status: student (n = 2), voluntary job (n = 1); clinician’s profession: kinesiologist (n = 1), sports scientist (n = 1), sports teacher (n = 2); FCE purpose: review work capacity for job duties suggestion (n = 1)

### Floor-to-Waist Lift Results

The simple multilevel regression analyses showed that 24 biopsychosocial variables were statistically significantly associated with floor-to-waist lifting performance (Online Resource 3). The final multilevel model included eight biological and psychological variables, these fixed effects explained 42.0% of total variance (Table 2). Altogether, greater weight lifted was associated with taller and male subjects, and with lower reported pain intensity, disability, effort during FCE test, and social isolation. Patients that performed to their maximum effort as observed by clinicians or who did not prematurely end the test lifted more weight. Clinician and country random effects composed approximately 39% of the total residual variance.

**Table 2:** Results from multiple multilevel regression analyses with FCE test performances as dependent variables are given; unstandardized regression coefficients and their standard errors (b(SE)), and model’s deviance (− 2 * LogLikelihood) by the addition of random and fixed effects are shown

	Floor-to-waist lift^a^ (n = 294)		Six minute walk^b^ (n = 224)		Right handgrip strength^c^ (n = 335)		Left handgrip strength^c^ (n = 336)	
	b (SE)	Deviance	b (SE)	Deviance	b (SE)	Deviance	b (SE)	Deviance
*Null model*		2213.26		2878.28		2847.88		2826.82
Random effects		2193.04***		2867.71**		2806.11***		2787.86***
Clinician and measurement country								
Fixed effects^d^		1941.21***		2643.19***		2590.43***		2597.43***
Intercept	− 2.01 (9.86)		219.87 (130.89)		− 3.67 (18.00)		− 18.96 (17.39)	
Age (years)					− 0.16 (0.06)		− 0.13 (0.05)	
Sex								
Female	− 4.97 (1.01)				− 11.33 (1.82)		− 11.38 (1.77)	
Height (cm)	0.17 (0.05)		1.98 (0.67)		0.34 (0.10)		0.38 (0.09)	
BMI (kg/m^2^)			− 3.82 (1.16)					
Affected body area								
Lower extremity					− 0.33 (2.01)			
Upper extremity					− 8.19 (2.32)			
Neck					− 5.94 (2.02)			
Generalized					− 5.62 (1.82)			
Other					9.17 (8.30)			
Observed physical effort								
Heavy	7.74 (1.28)							
Maximum	9.04 (1.70)							
Test ended prematurely								
Yes	− 4.87 (1.19)		− 168.60 (24.50)					
Post-test HR (bpm)			1.99 (0.30)					
Reported pain intensity (NRS)	− 0.64 (0.21)		− 10.89 (2.81)		− 0.77 (0.33)		− 0.91 (0.28)	
Reported effort during FCE test (Borg CR-10)	− 0.61 (0.20)		− 20.49 (2.79)					
Reported social isolation	− 0.34 (0.17)							
Reported catastrophizing					− 0.61 (0.27)			
Reported disability (PDI)	− 0.07 (0.04)							
Physical work demands (DOT)								
Light					0.65 (2.38)		0.93 (2.31)	
Medium					− 0.06 (2.24)		0.78 (2.19)	
Heavy					6.77 (2.51)		6.26 (2.43)	
Very heavy					2.66 (2.86)		5.65 (2.77)	
Days off work								
Less than ¼ year			3.57 (22.30)					
¼–½ year			− 59.66 (22.75)					
½–1 year			− 12.31 (22.18)					
1–2 years			− 27.40 (25.08)					
More than 2 years			− 89.19 (24.17)					

Reference category: patient’s affected body area: low back; observed physical effort by clinicians: light to moderate; patient’s physical work demands: sedentary; patient’s days off work: no days off

*BMI* Body Mass Index, *HR* heart-rate, *NRS* Numeric Rating Scale, *PDI* Pain Disability Index, *DOT* Dictionary of Occupational Titles, *FCE* Functional Capacity Evaluation

Significance: * < 0.05; ** < 0.01; *** < 0.001

^a^Measured in kg

^b^Measured in m

^c^Measured in kgF

^d^Each of the fixed effects factors showed a significant improvement of the model at its addition

### Six-Minute Walk Results

The associations between 6MWT performance and 19 of the collected biopsychosocial variables were statistically significant (Online Resource 3). The final multilevel model included seven biopsychosocial variables, these fixed effects explained 64.6% of total variance (Table 2). Altogether, longer distances covered were related to participants that were taller, had lower BMI, higher post-test HR, and patients reporting lower pain intensity and effort during FCE test. Those that were out of work for 3 months and more, or ended the test prematurely showed lower walking performance. Clinician and country random effects composed approximately 1% of the total residual variance.

### Handgrip Strengths Results

A total of 14 biopsychosocial variables were significantly associated with right handgrip strength test and 12 with left handgrip strength test. Both handgrip strength performances showed statistically significant associations with similar characteristics, differing only in the association of right handgrip performance with patient-reported anxiety and depression scores (Online Resource 3). The final models included seven biopsychosocial variables for right handgrip and five for left handgrip. These fixed effects of these models explained 38.6 and 39.8% of total variance, respectively (Table 2). Altogether, patients who were younger, taller, and male, with reported lower pain intensity and catastrophizing scores performed better on handgrip strength tests. Also, if they were not affected by upper or lower extremity, neck or generalized pain, or their work physical demands were light, heavy or very heavy, their handgrip strength was greater. Clinician and country random effects composed approximately 25% of the total residual variance for right handgrip, and 19% for left handgrip.

## Discussion

This international cross-sectional study was performed to examine what biopsychosocial factors were associated with FCE results in patients with painful musculoskeletal conditions, with a focus on social factors across multiple countries. Patients’ sex, height, reported pain intensity, effort during FCE test, social isolation, and disability, clinician’s observed physical effort, and whether FCE test was prematurely ended were associated with lift test results. Patient’s height, BMI, post-test HR, their reported pain intensity and effort during FCE test, days off work, and whether FCE test was prematurely ended were associated with walk test results. Patient’s age, sex, height, affected body area, reported pain intensity and catastrophizing, and physical work demands were associated with handgrip test results. All these results were independently associated with FCE performance when considering dependency of measurements within clinician and country. Overall, biopsychosocial factors were consistently associated with performance on every FCE test, although factors differed between tests.

Compared to a previous study performed in three different countries [18], the present study included a larger number of countries spread worldwide to study the association of FCE tests with a comprehensive quantity of variables, including a variety of patients, clinicians, and settings, which makes our results unique. The data collected in eight participating countries has produced a large sample of patients with chronic musculoskeletal pain who differ in physical, personal and social characteristics, including system and FCE characteristics. This heterogeneity in sample characteristics reveals a more profound understanding of the reasons for the differences in FCE results.

The important finding in the current study is that biopsychosocial factors contributed significantly to the models. Literature on FCE shows evidence of biological and psychological factors’ association with FCE, while research on social factors is limited [46]. The influence of social and/or environmental factors within the occupational field on individual’s functioning and disability due to conditions such as low back pain has been stressed [47, 48]. This is supported in the present study where social factors were found to be related to all FCE tests. Specifically, all FCE tests were significantly related to the healthcare environment in which the measurements were taken. The clinician and the measurement country were both significantly associated with all FCE test results, the latter result being supported by previous research [18].

It is noteworthy to state that simple regression models showed that participant’s cultural background as measured by nationality and/or mother language were also significantly related to three of the tests (lift and handgrip strength tests). Although these variables were not included in the final regression models due to the risk of multicollinearity with the measurement country, their associations should not be overlooked. Such associations may relate differences in FCE performance to the cultural background of the patients, in agreement with previous research [16, 17].

Taking a closer look at the relationship between clinicians’ characteristics and FCE tests performance, it is seen that the clinician is significantly related, but clinician fear beliefs were not associated. Former studies on lift capacity as assessed by clinicians with different levels of fear-avoidance behavior showed differences in lifting performance in healthy young participants [19]. A plausible explanation for this difference may be that the current study was performed with patients with chronic musculoskeletal pain, and that the scores obtained with the pain beliefs questionnaire were similar across clinicians regardless of their professional experience (3.5–45 years). In contrast to the absence of influence of clinician’s psychological characteristics, the observed physical effort during lifting capacity remained in the final model. This further supports the importance of clinician’s observation as a way to determine patient’s effort levels, in accordance with a previous study that validated this tool [49].

Analogous to studies performed within a specific societal context, the present research has identified relevant associations of patient-reported psychological factors with FCE results. The information collected from patients has shown that pain intensity was associated with all FCE tests. Patient-reported disability and social isolation were associated with weight lifted; patient-reported effort was associated with both lifted weight and walked distance; while patient-reported catastrophizing was associated with right handgrip strength. Although previous research supports these findings, some variables expected to be associated with FCE results were missing in the final models. Patient-reported anxiety, depression, fear of movement, secondary gain or compensation, and disability were not associated with walk distance or handgrip strength, patient-reported catastrophizing was not associated with left handgrip strength, all of which are not consistent with previous systematic and literature reviews [46, 50], or opinions of scientists, clinicians, and patients [10]. This study does, therefore, provide a new perspective of the magnitude of the psychological factors associated with FCE tests.

A major strength of this study is the heterogeneity of patients’ social context characteristics. A plural sample such as the one created for this study with the large number of variables investigated, allows for greater external validity of the results. However, this added to complexity of variable measurement due to differences in legislation, policy and definitions across countries.

The study also has some limitations. First, due to the international nature of the study, English was not an official language for some participating countries. In those cases, liaisons were in charge of finding valid translations of the questionnaires or of translating them into one of the official languages of the country. These translations’ validity was not tested, and the extent to which this could produce bias is not known. Second, for the purpose of measuring clinician’s pain beliefs, BBQ was used. The questionnaire has been used and tested in the general population, including people with and without back pain and clinical students (nursing students) [41, 42]. Therefore, we believe that it is unlikely that the BBQ may have introduced bias to the results of this study. Additionally, such questionnaire was adapted in the wording, ‘back trouble’ was changed into ‘musculoskeletal pain’. Again, its validity was not tested but we assumed that no important bias was introduced through this minor change. Third, to ensure that the samples would be representative of the population undergoing FCE in each of the participating countries, more than one facility per country was asked to participate. However, we cannot be completely certain that our sample is generalizable to the participating countries. Fourth, some countries and facilities willing to participate experienced methodological, recruitment or time barriers, which led to their exclusion from the study. Nevertheless, from our point of view, the sample varies sufficiently as to allow the generalization of results. Fifth, only direct effects were tested on FCE test performances. Future research may use structural equation modeling to describe indirect effects and causal pathways. Finally, the categorization of biopsychosocial factors is not beyond debate. For the present study the division of bio-psycho-social factors was made within the ICF framework [51]. Nevertheless, readers can, based on the tables, report different choices. This would change the distribution of explained variance among the three types of factors, but not the explained variance of the full models.

Our findings bring some implications in the assessment of individuals undergoing FCEs. The large sample size, the heterogeneity of patients, and the plethora of measured factors, make this study unique and puts the results obtained so far into perspective. Many of the previously expected and studied associations of biopsychosocial factors with FCE within a certain societal context have been supported. However, other biopsychosocial factors as anxiety, depression, fear of movement, secondary gain or compensation, or clinician’s fear beliefs during lift test, have been shown to conflict with previous studies. Around 40% of the variance was explained in lift and handgrip models while for the walk model, it was 60%, thus, there is still a large proportion that remains unexplained. Additionally, the correlation among participants within clusters (within clinician and country) was between 18 and 39% in the lift and handgrip models and 1% in the walk model, but an explanation on the mechanism of such association is so far unknown. This further knowledge of the association of the context in which individuals perform FCEs and their results, supports the importance of the biopsychosocial framework. More evidence with a similar design as the current one is needed to disentangle nature of the associations of individuals’ context with the measurements, to identify the factors that remain unknown, and to allow for the generalization and for a more balanced interpretation of results, which in time will empower not only the FCE, but also other similar instruments.

## Conclusion

Biopsychosocial factors were associated with performance on every FCE test across multiple countries; specifically patients’ height, reported pain intensity, clinician, and measurement country. Social factors, which had been under-researched, showed a consistent association with FCE performances. This supports the concept of considering patients from a biopsychosocial perspective in combination with different social contexts, and allows for the generalizability of the findings. Further research to replicate these results and to increase our understanding of the differences across the different societal contexts is needed.

## Electronic supplementary material

Below is the link to the electronic supplementary material.

Supplementary material 1 (PDF 166 KB)

Supplementary material 2 (PDF 491 KB)

Supplementary material 3 (PDF 198 KB)
