# Correction to: Functional Capacity Evaluation in Different Societal Contexts: Results of a Multicountry Study

**DOI:** 10.1007/s10926-018-9797-3

**Published:** 2018-06-26

**Authors:** Jone Ansuategui Echeita, Matthias Bethge, Berry J. van Holland, Douglas P. Gross, Jan Kool, Peter Oesch, Maurizio A. Trippolini, Elizabeth Chapman, Andy S. K. Cheng, Robert Sellars, Megan Spavins, Marco Streibelt, Peter van der Wurff, Michiel F. Reneman

**Affiliations:** 1 Department of Rehabilitation Medicine, University Medical Center Groningen, University of Groningen, P.O. Box 30.002, 9750 RA Haren, Groningen, The Netherlands; 2 Institute of Social Medicine and Epidemiology, University of Lübeck, Lübeck, Germany; 3 Institute for Sports Studies, Hanze University of Applied Sciences, Groningen, The Netherlands; 4 Department of Physical Therapy, University of Alberta, Edmonton, AB Canada; 5 Rehabilitation Centre Valens, Valens, Switzerland; 6 Center for Disability Research, Liberty Mutual Research Institute for Safety, Hopkinton, Boston, USA; 7 PhD in Rehabilitation Sciences Program, Institute for Health Professions, Massachusetts General Hospital (MGH), Charlestown, Boston, USA; 8 Department of Work Rehabilitation, Rehaklinik Bellikon, Suva Care, Bellikon, Switzerland; 9 Toyota Motor Manufacturing Canada, Puslinch, ON Canada; 10 Ergonomics and Human Performance Laboratory, Department of Rehabilitation Sciences, The Hong Kong Polytechnic University, Hong Kong, China; 11 FCE Systems Ltd, Geraldine, New Zealand; 12 Occupational Therapy Inc., Randburg, South Africa; 13 Department of Rehabilitation, German Federal Pension Insurance, Berlin, Germany; 14 Research & Development, Military Rehabilitation Center Aardenburg, Doorn, The Netherlands; 15 Institute for Human Movement Studies, HU University of Applied Sciences, Utrecht, The Netherlands

## Correction to: Journal of Occupational Rehabilitation 10.1007/s10926-018-9782-x

The original version of this article unfortunately contained a mistake in Table 2. The data under column head “Left handgrip strength (n = 336)” was erroneously omitted during the production process. The corrected Table 2 is given below.

The original article has been corrected.

**Table 2:** Results from multiple multilevel regression analyses with FCE test performances as dependent variables are given; unstandardized regression coefficients and their standard errors (b(SE)), and model’s deviance (− 2 * LogLikelihood) by the addition of random and fixed effects are shown

	Floor-to-waist lift^a^ (n = 294)		Six minute walk^b^ (n = 224)		Right handgrip strength^c^ (n = 335)		Left handgrip strength^c^ (n = 336)	
	b (SE)	Deviance	b (SE)	Deviance	b (SE)	Deviance	b (SE)	Deviance
*Null model*		2213.26		2878.28		2847.88		2826.82
Random effects		2193.04***		2867.71**		2806.11***		2787.86***
Clinician and measurement country								
Fixed effects^d^		1941.21***		2643.19***		2590.43***		2597.43***
Intercept	− 2.01 (9.86)		219.87 (130.89)		− 3.67 (18.00)		− 18.96 (17.39)	
Age (years)					− 0.16 (0.06)		− 0.13 (0.05)	
Sex								
Female	− 4.97 (1.01)				− 11.33 (1.82)		− 11.38 (1.77)	
Height (cm)	0.17 (0.05)		1.98 (0.67)		0.34 (0.10)		0.38 (0.09)	
BMI (kg/m^2^)			− 3.82 (1.16)					
Affected body area								
Lower extremity					− 0.33 (2.01)			
Upper extremity					− 8.19 (2.32)			
Neck					− 5.94 (2.02)			
Generalized					− 5.62 (1.82)			
Other					9.17 (8.30)			
Observed physical effort								
Heavy	7.74 (1.28)							
Maximum	9.04 (1.70)							
Test ended prematurely								
Yes	− 4.87 (1.19)		− 168.60 (24.50)					
Post-test HR (bpm)			1.99 (0.30)					
Reported pain intensity (NRS)	− 0.64 (0.21)		− 10.89 (2.81)		− 0.77 (0.33)		− 0.91 (0.28)	
Reported effort during FCE test (Borg CR-10)	− 0.61 (0.20)		− 20.49 (2.79)					
Reported social isolation	− 0.34 (0.17)							
Reported catastrophizing					− 0.61 (0.27)			
Reported disability (PDI)	− 0.07 (0.04)							
Physical work demands (DOT)								
Light					0.65 (2.38)		0.93 (2.31)	
Medium					− 0.06 (2.24)		0.78 (2.19)	
Heavy					6.77 (2.51)		6.26 (2.43)	
Very heavy					2.66 (2.86)		5.65 (2.77)	
Days off work								
Less than ¼ year			3.57 (22.30)					
¼–½ year			− 59.66 (22.75)					
½–1 year			− 12.31 (22.18)					
1–2 years			− 27.40 (25.08)					
More than 2 years			− 89.19 (24.17)					

Reference category: patient’s affected body area: low back; observed physical effort by clinicians: light to moderate; patient’s physical work demands: sedentary; patient’s days off work: no days off

*BMI* Body Mass Index, *HR* heart-rate, *NRS* Numeric Rating Scale, *PDI* Pain Disability Index, *DOT* Dictionary of Occupational Titles, *FCE* Functional Capacity Evaluation

Significance: * < 0.05; ** < 0.01; *** < 0.001

^a^Measured in kg

^b^Measured in m

^c^Measured in kgF

^d^Each of the fixed effects factors showed a significant improvement of the model at its addition

