# Supplementary material for: Functional Capacity Evaluation in Different Societal Contexts: Results of a Multicountry Study
Source: J Occup Rehabil. 2018 May 25;29(1):222–36. doi: 10.1007/s10926-018-9782-x (PMC6510856; doi:10.1007/s10926-018-9782-x)
Supplement: Supplementary file 1 — Supplementary material 1 (PDF 166 KB) [file 10926_2018_9782_MOESM1_ESM.pdf]

ELECTRONIC SUPPLEMENTARY MATERIAL

Title: Functional Capacity Evaluation in different societal contexts: Results of a multicountry study.

Journal: Journal of occupational rehabilitation.

Authors: Jone Ansuategui Echeita, Matthias Bethge, Berry J. van Holland, Douglas P. Gross, Jan Kool, Peter Oesch, Maurizio A. Trippolini, Elizabeth Chapman, Andy S.K. Cheng, Robert Sellars, Megan Spavins, Marco Streibelt, Peter van der Wurff, Michiel F. Reneman.

Corresponding Author: Jone Ansuategui Echeita, University of Groningen, University Medical Center Groningen, Department of Rehabilitation Medicine, Groningen, The Netherlands. Email: j.ansuategui.echeita@umcg.nl

Online Resource 1 System characteristics of the countries participating in the study.

	System Characteristics
**The Netherlands**	Inhabitants of the Netherlands are insured for medical care by law. In case of sick-leave, employees are fully compensated during their first year on sick-leave, and at 70% of their last salary on the second year; the employer is responsible for these costs. After that period, the government provides a maximum compensation of 70% of their last salary for two years for the loss of work. If the employers do not collaborate sufficiently on worker's reintegration during the first two years of sick-leave, they are responsible of the costs (not the government). Afterwards, the government supplies a compensation which may not reach the 70% of their last salary (minimum subsistence).
**Canada**	In Canada, a variety of compensation programs exist to support residents in case of sickness or disability, these are both private and public insurance systems. The majority of employers are legally obligated to offer workers' compensation benefits through Workers' Compensation Boards. These programs offer economic support and healthcare services for workers injured during the course of their employment. Many companies and individuals also purchase supplemental disability insurance in case of non-work related disability or sickness. Disability benefits are also offered publicly through the Federal government (Canada Pension Plan Disability Benefits), reimbursing up to 66% of their previous salary for up to two years if they are unable to work in their previous occupation. Following the two year mark, the employee will continue to receive benefits if they cannot complete any occupation. All of these different programs, agencies, and systems have unique policies and procedures.
**Switzerland**	Residents in Switzerland are insured by the Swiss Accident Insurance Fund on occupational and non-occupational accidents. In case of sickness mandatory health insurance covers costs for health care. All persons resident or gainfully employed in Switzerland are compulsorily insured against disability. Insured persons are entitled to disability insurance benefits if, as a result of a health defect, they partially or fully lose their ability to be gainfully employed. Injured individuals receive a compensation of up to 80 % of their previous salary, medical and vocational assistance up to a maximum of 2 years, and disability pensions due to injury. Many patients with chronic conditions become unemployed. Unemployed individuals receive 70% of the insured income for a maximum period of two years; this can reach 80% if they have dependent children or their unemployment benefit does not reach the minimum level.
**Germany**	Residents in Germany pay compulsory contributions to a Pension Insurance Agency and to a Health Insurance Agency. In the case of sickness absence, employees are paid a regular wage by their employer for 6 weeks. Afterwards, the Health Insurance Agency is in charge of the sickness benefits for a maximum of 72 weeks at the 70 % of the gross income. In the case of permanent work disability, the Pension Insurance Agency may approve either a disability pension or the use of rehabilitation services to avoid disability pensions. The disability pension depends on the contributions the worker has paid until that moment. The sickness benefits during rehabilitation are of 68 % of their last net income, rising up to 75 % for those patients with children.

**Austria**	In Austria the social system is regulated by the state and administered regionally. Workers, employees and self-employed persons pay compulsory contributions to the Austrian Social Insurance. In case of sickness absence workers and employees get paid 100% of their salaries by their employers for at least 6 weeks, which could be prolonged if it is regulated in a work agreement of the company. After that period they receive sickness benefit at the 75%-80% of the former salary as regulated by the ASVG-law (General Social Insurance Act). In case of accidents at work, the workers receive financial compensation, and medical and occupational assistance. These work accident-related compensations are paid for by the AUVA (General Accident Insurance Institute) for employees and by the SVA-Insurance (Austrian Social Insurance Authority for Business) for self-employed workers. Unemployed people receive 55% of their last net salary in addition to family allowances by the AMS (Austrian Public Employment Service).
**South Africa**	South Africa has both public and private disability insurance systems. The South African Social Security Agency (SASSA) serves the majority of the population but the disability benefits are limited. Individuals with such a disability as to be unfit for work for more than six months may get a temporary grant for a period of six to 12 months, or a permanent grant for more than 12 months (this does not imply that it will be for life). Those individuals who can afford private disability insurance, are generally suitable for a disability benefit of 75% of their previous salary, although this may vary according to the insurance provider and the type of disability insurance purchased. Also, a lump sum benefit is available, but usually at a lower premium than the monthly income replacement type of benefit.
**New Zealand**	New Zealanders and visitors are covered by the Accident and Compensation Commission (‘ACC’) for treatment of accidents from all causes (work and non-work injuries). ACC runs on a 'no fault' scheme and also provides most case management. Compensation is of 80% of pre-injury earnings to NZ citizens or permanent residents who are employed at the time of accident. There are other 'third party providers' and 'self-insured' companies who run their own schemes for workplace injuries; nevertheless, these are still bound by ACC legislation.
**Hong Kong , China**	The employee compensation system for work injuries is a no-fault, non-contributory system, regulated by the Employee´s Compensation Ordinance. This Ordinance covers all local (Hong Kong) employment contracts of service or apprenticeship, also while working outside of Hong Kong. In the event of a work accident resulting in injury to a worker, the employer will be liable to pay for medical expenses, compensation for permanent total incapacity suffered by the worker, and periodic payments (80% of pre-injury earnings) during the period of temporary incapacity up to 24 months. Assessment of the necessary period of absence and the loss of earning capacity is provided for by the Employees’ Compensation Boards of the Hong Kong Labor Department.
