# Supplementary material for: Functional Capacity Evaluation in Different Societal Contexts: Results of a Multicountry Study
Source: J Occup Rehabil. 2018 May 25;29(1):222–36. doi: 10.1007/s10926-018-9782-x (PMC6510856; doi:10.1007/s10926-018-9782-x)
Supplement: Supplementary file 2 — Supplementary material 2 (PDF 491 KB) [file 10926_2018_9782_MOESM2_ESM.pdf]

ELECTRONIC SUPPLEMENTARY MATERIAL

Title: Functional Capacity Evaluation in different societal contexts: Results of a multicountry study.

Journal: Journal of occupational rehabilitation.

Authors: Jone Ansuategui Echeita, Matthias Bethge, Berry J. van Holland, Douglas P. Gross, Jan Kool, Peter Oesch, Maurizio A. Trippolini, Elizabeth Chapman, Andy S.K. Cheng, Robert Sellars, Megan Spavins, Marco Streibelt, Peter van der Wurff, Michiel F. Reneman.

Corresponding Author: Jone Ansuategui Echeita, University of Groningen, University Medical Center Groningen, Department of Rehabilitation Medicine, Groningen, The Netherlands. Email: j.ansuategui.echeita@umcg.nl

Online Resource 2 Patients' and clinicians' characteristics per country; mean ± SD or n (%) are shown.

				NL	CA	CH	DE	AT	ZA	NZ	CN
Floor-to-Waist Lift Performance (kg)				23.8 ± 12.7	9.9 ± 7.5	19.4 ± 8.5	19.1 ± 8.8	23.5 ± 4.5	12.7 ± 8.7	20.3 ± 12.8	12.4 ± 5.7
Six Minute Walk Test Performance (m)				514.7 ± 122.7	468.0 ± 97.5	535.6 ± 123.5	393.8 ± 166.8	547.0 ± 67.4	413.7 ± 169.7	458.1 ± 173.7	361.8 ± 89.0
Right Handgrip Strength Performance (kgF)				34.1 ± 14.2	26.6 ± 15.4	38.4 ± 13.5	34.2 ± 13.9	35.3 ± 14.4	27.1 ± 13.3	48.0 ± 23.4	25.5 ± 7.7
Left Handgrip Strength Performance (kgF)				32.0 ± 13.6	22.0 ± 15.0	36.0 ± 13.3	33.0 ± 13.0	37.4 ± 12.0	23.5 ± 14.7	46.3 ± 21.6	26.1 ± 8.2
**Biological Factors**											
Patient's	Participating Patients			52	5	86	100	12	34	64	19
	Age (years)			42.3 ± 12.0	39.2 ± 9.6	40.7 ± 13.2	46.8 ± 11.8	41.0 ± 12.1	40.3 ± 10.7	45.8 ± 12.5	50.5 ± 10.9
	Sex (female)			32 (61.5%)	4 (80%)	28 (32.6%)	46 (46%)	0 (0%)	17 (50%)	20 (31.3%)	9 (47.4%)
	Height (cm)			174.6 ± 10.4	168.0 ± 5.8	174.3 ± 7.2	174.5 ± 8.8	181.7 ± 7.5	167.2 ± 11.7	173.9 ± 9.5	163.5 ± 6.1
	Weight (kg)			81.2 ± 16.6	65.6 ± 16.7	80.4 ± 17.4	86.1 ± 18.4	96.1 ± 24.6	78.9 ± 15.6	88.6 ± 19.5	58.2 ± 9.3
	BMI (kg/m^2)			26.5 ± 4.4	23.2 ± 5.6	26.4 ± 5.0	28.2 ± 5.5	29.0 ± 7.0	28.4 ± 5.9	29.2 ± 5.7	21.7 ± 2.8
	Affected Body Area	Low Back		19 (36.5%)	1 (20%)	27 (31.4%)	43 (43%)	0 (0%)	11 (32.3%)	22 (34.4%)	10 (52.6%)
		Lower Extremity		1 (1.9%)	0 (0%)	15 (17.4%)	11 (11%)	6 (50%)	7 (20.6%)	18 (28.1%)	8 (42.1%)
		Upper Extremity		2 (3.8%)	0 (0%)	9 (10.5%)	4 (4%)	5 (41.7%)	5 (14.7%)	16 (25%)	1 (5.3%)
		Neck		14 (27%)	2 (40%)	14 (16.3%)	17 (17%)	0 (0%)	3 (8.8%)	3 (4.7%)	0 (0%)
		Generalized		16 (30.8%)	2 (40%)	21 (24.4%)	25 (25%)	1 (8.3%)	7 (20.6%)	4 (6.3%)	0 (0%)
		Other		0 (0%)	0 (0%)	0 (0%)	0 (0%)	0 (0%)	1 (2.9%)	1 (1.6%)	0 (0%)
	Floor-to-Waist Lift Test										
		Observed Physical Effort	Light to Moderate	12 (23.1%)	4 (80%)	10 (11.6%)	7 (7%)	0 (0%)	8 (23.5%)	49 (76.6%)	2 (10.5%)
			Heavy	26 (50.0%)	0 (0%)	11 (12.8%)	39 (39%)	0 (0%)	4 (11.8%)	14 (21.9%)	12 (63.2%)
			Maximum	14 (26.9%)	1 (20%)	63 (73.3%)	54 (54%)	12 (100%)	20 (58.8%)	1 (1.6%)	5 (26.3%)
		Test Ended Prematurely (yes)		25 (48.1%)	2 (40%)	21 (24.4%)	46 (46%)	0 (0%)	8 (23.5%)	22 (34.4%)	0 (0%)
	Six-Minute Walk Test										
		Test Prematurely Ended (yes)		0 (0%)	0 (0%)	9 (11.3%)	1 (1%)	0 (0%)	2 (5.9%)	6 (9.4%)	0 (0%)
**Psychological Factors**											
Patient-	Pain Intensity (NRS) (0-10)			5 (2-7)	7 (4-8)	4.5 (3-6)	6 (4-7)	3 (1.3-5)	8 (5-9)	5 (3-6.8)	5 (3-5)

			NL		CA		CH		DE		AT		ZA		NZ		CN	
Reported	Pain Duration	Less than ½ year	9	(17.3%)	0	(0%)	16	(18.6%)	23	(23%)	5	(41.7%)	2	(5.9%)	6	(9.4%)	3	(15.8%)
		½ - 1 year	12	(23.1%)	3	(60%)	25	(29.1%)	30	(30%)	3	(25%)	2	(5.9%)	10	(15.6%)	10	(52.6%)
		1 - 2 years	11	(21.2%)	1	(20%)	15	(17.4%)	15	(15%)	3	(25%)	5	(14.7%)	13	(20.3%)	6	(31.6%)
		2 - 3 years	7	(13.5%)	0	(0%)	6	(7.0%)	21	(21%)	1	(8.3%)	9	(26.5%)	10	(15.6%)	0	(0%)
		3 - 10 years	7	(13.5%)	1	(20%)	16	(18.6%)	9	(9%)	0	(0%)	12	(35.3%)	19	(29.7%)	0	(0%)
		More than 10 years	5	(9.6%)	0	(0%)	7	(8.1%)	2	(2%)	0	(0%)	0	(0%)	6	(9.4%)	0	(0%)
	Effort during Floor-to-Waist Lift Test (Borg CR-10)		7.4 ±	1.9	5.4 ±	2.4	6.5 ±	1.8	7.0 ±	2.0	5.8 ±	1.9	8.3 ±	1.6	4.8 ±	2.5	5.7 ±	1.8
	Effort during Six-Minute Walk Test (Borg CR-10)		4.4 ±	2.2	6.0 ±	0.7	4.5 ±	2.3	6.3 ±	1.7	4 ±	1.9	6.4 ±	2.3	4.3 ±	2.9	4.3 ±	2.1
	Screening Questionnaire	Anxiety (0-10)	2	(1-5)	4	(2-9.5)	1	(0-5)	3	(1-5)	0.5	(0-2.5)	5.5	(3-8)	4	(2-7)	6	(5-7)
		Social Isolation (0-10)	1	(1-3)	6	(4-7.5)	0.5	(0-3)	2	(0-4.8)	0	(0-0)	6	(1.8-9)	4	(1-6)	5	(3-6)
		Catastrophizing (0-10)	3.5	(1.5-6)	4	(2.5-8.5)	3.5	(1.5-6)	5.5	(3.5-7.4)	1	(0.5-4)	7.8	(3.4-9)	4.5	(2-7)	5.5	(3-7)
		Depression (0-10)	3	(1.5-5.5)	4	(2.3-8.3)	3.5	(1.5-6)	5	(2-6)	0	(0-1.8)	7.3	(3.4-9)	4	(2-6.5)	4.5	(3-6)
		Fear of Movement (0-10)	4	(2-6.5)	4	(1.3-6)	3	(1-5.5)	5	(3.5-6.5)	0.5	(0-1)	7.5	(5-9.6)	4.8	(3-7.9)	4.5	(2.5-5)
	Self-Reported Disability (PDI) (0-70)		29.5	(20-45.5)	43	(16-54.5)	28.5	(20.8-36.5)	29	(15-39)	16.5	(7-22.5)	43	(27.8-53.3)	34	(23.3-46.8)	30	(21-37)
	Family / Home Responsibilities (0-10)		5	(2-7.5)	7	(1-8)	4.5	(3-6.3)	5	(3-7)	4	(1.3-5)	6	(4-8)	5	(3-7)	5	(3-6)
	Recreation (0-10)		6	(3-8)	7	(1-8.5)	6	(3-8)	5	(3-7.3)	2	(1.3-3.8)	8	(5-9.3)	7	(4-8)	5	(5-5)
	Social Activity (0-10)		5	(1-8)	7	(3.5-9)	4	(2-6)	4	(1-6)	1.5	(0.3-2.8)	6	(3.8-8.3)	5.5	(3-7)	4	(3-5)
	Occupation (0-10)		6	(5-8)	8	(2.5-10)	7	(5-9)	6	(3.3-8)	5	(1.3-7.5)	7	(4-9)	7	(5-8)	5	(3-6)
	Sexual Behaviour (0-10)		3	(0.8-6)	5	(4-7)	4	(1-6)	4	(1-5)	0	(0-1.8)	7	(2.8-8)	4	(2-8)	4	(0-6)
	Self-Care (0-10)		2	(0-4.8)	4	(1-7)	2	(0-4)	2	(0-4)	0	(0-1.8)	5	(2-7.3)	5	(2-6)	4	(2-5)
	Life-Support Activity (0-10)		2.5	(0-5)	5	(0.5-7.5)	1	(0-3.3)	2	(0-4)	0	(0-2)	5	(1.8-7)	2	(0.3-5)	4	(3-5)
	Work Ability Index – Single Item (WAS) (0-10)		5	(3-6)	3	(1-6.5)	4	(2-6)	4	(2-6)	7	(5.3-8)	4	(0.8-6)	3	(2-5)	5	(4-7)
**Social Factors**																		
Patient's	Living Country	Same as Measurement Country	52	(100%)	5	(100%)	86	(100%)	100	(100%)	12	(100%)	34	(100%)	64	(100%)	19	(100%)
		Other	0	(0%)	0	(0%)	0	(0%)	0	(0%)	0	(0%)	0	(0%)	0	(0%)	0	(0%)
	Cultural background [b]	Same as Measurement Country	51	(98.1%)	4	(80%)	59	(68.6%)	92	(92%)	9	(75%)	34	(100%)	55	(85.9%)	19	(100%)
		Other	1	(1.9%)	1	(20%)	27	(31.4%)	8	(8%)	3	(25%)	0	(0%)	9	(14.1%)	0	(0%)
	Mother Language [c]	Official Language in Country	51	(98.1%)	4	(80%)	76	(88.4%)	94	(94%)	8	(66.7%)	34	(100%)	61	(95.3%)	19	(100%)
		Other	1	(1.9%)	1	(20%)	10	(11.6%)	6	(6%)	4	(33.3%)	0	(0%)	3	(4.7%)	0	(0%)
	Marital status	Single	12	(23.1%)	1	(20%)	39	(45.3%)	20	(20%)	3	(25%)	14	(41.2%)	9	(14.1%)	0	(0%)
		Living Together	7	(13.5%)	0	(0%)	0	(0%)	0	(0%)	0	(0%)	0	(0%)	0	(0%)	0	(0%)
		Married	28	(53.8%)	3	(60%)	31	(36.0%)	59	(59%)	5	(41.7%)	15	(44.1%)	38	(59.4%)	18	(94.7%)
		Common Law	0	(0%)	1	(20%)	0	(0%)	4	(4%)	3	(25%)	0	(0%)	6	(9.4%)	0	(0%)
		Separated	0	(0%)	0	(0%)	0	(0%)	0	(0%)	0	(0%)	4	(11.8%)	2	(3.1%)	0	(0%)
		Divorced	4	(7.7%)	0	(0%)	14	(16.3%)	14	(14%)	1	(8.3%)	1	(2.9%)	7	(10.9%)	1	(5.3%)
		Widowed	1	(1.9%)	0	(0%)	2	(2.3%)	3	(3%)	0	(0%)	0	(0%)	2	(3.1%)	0	(0%)
	Educational Level	No Degree	0	(0%)	0	(0%)	0	(0%)	2	(2%)	0	(0%)	0	(0%)	6	(9.4%)	0	(0%)
		Elementary Education	3	(5.8%)	1	(20%)	10	(11.6%)	0	(0%)	3	(25%)	1	(2.9%)	13	(20.3%)	4	(21.1%)
		High-school Education	10	(19.2%)	0	(0%)	15	(17.4%)	36	(36%)	0	(0%)	22	(64.7%)	20	(31.3%)	15	(78.9%)
		Vocational Training	27	(51.9%)	2	(40%)	59	(68.6%)	56	(56%)	6	(75%)	8	(23.5%)	18	(28.1%)	0	(0%)
		Bachelor	9	(17.3%)	1	(0%)	1	(1.2%)	5	(5%)	0	(0%)	3	(8.8%)	7	(10.9%)	0	(0%)
		Master	2	(3.8%)	1	(20%)	1	(1.2%)	1	(1%)	0	(0%)	0	(0%)	0	(0%)	0	(0%)
		Doctorate	1	(1.9%)	0	(0%)	0	(0%)	0	(0%)	0	(0%)	0	(0%)	0	(0%)	0	(0%)
	Physical Work Demands (DOT)	Sedentary	13	(25.0%)	1	(20%)	10	(11.6%)	7	(7%)	0	(0%)	8	(23.5%)	5	(7.8%)	1	(5.3%)
		Light	8	(15.4%)	2	(20%)	15	(17.4%)	22	(22%)	0	(0%)	11	(32.4%)	12	(18.8%)	7	(36.8%)
		Medium	19	(36.5%)	2	(40%)	18	(20.9%)	39	(39%)	3	(25%)	5	(14.7%)	27	(42.2%)	4	(21.1%)
		Heavy	10	(19.2%)	0	(0%)	21	(24.4%)	25	(25%)	7	(58.3%)	6	(17.6%)	16	(25%)	4	(21.1%)

			NL		CA		CH		DE		AT		ZA		NZ		CN	
		Very Heavy	2	(3.8%)	0	(0%)	22	(25.6%)	7	(7%)	2	(16.7%)	0	(0%)	4	(6.3%)	3	(15.8%)
	WorkStatus	Working on Regular Duty	14	(26.9%)	0	(0%)	7	(8.1%)	27	(27%)	0	(0%)	9	(26.5%)	2	(3.1%)	0	(0%)
		Working on Modified Duty	8	(15.4%)	0	(0%)	1	(1.2%)	6	(6%)	0	(0%)	3	(8.8%)	13	(20.3%)	1	(5.3%)
		Working on Reduced Duty	11	(21.2%)	1	(20%)	8	(9.3%)	0	(0%)	0	(0%)	0	(0%)	2	(3.1%)	0	(0%)
		On Full Sick-Leave	16	(30.8%)	0	(0%)	58	(67.4%)	67	(67%)	8	(66.7%)	13	(38.2%)	4	(6.3%)	17	(89.5%)
		Unemployed	1	(1.9%)	2	(40%)	7	(8.1%)	0	(0%)	4	(33.3%)	9	(26.5%)	27	(42.2%)	1	(5.3%)
		On Disability Allowance	2	(3.8%)	2	(40%)	4	(4.7%)	0	(0%)	0	(0%)	0	(0%)	14	(21.9%)	0	(0%)
		Other	0	(0%)	0	(0%)	1	(1.2%)	0	(0%)	0	(0%)	0	(0%)	2	(3.1%)	0	(0%)
	Days Off Work	No days off	24	(46.2%)	0	(0%)	9	(10.5%)	27	(27%)	0	(0%)	1	(2.9%)	3	(4.7%)	0	(0%)
		Less than ¼ year	12	(23.1%)	1	(20%)	23	(26.7%)	13	(13%)	0	(0%)	7	(20.6%)	4	(6.3%)	0	(0%)
		¼ - ½ year	6	(11.5%)	0	(0%)	18	(20.9%)	22	(22%)	5	(41.7%)	6	(17.6%)	9	(14.1%)	3	(15.8%)
		½ - 1 year	6	(11.5%)	4	(80%)	17	(19.8%)	25	(25%)	3	(25%)	6	(17.6%)	14	(21.9%)	10	(52.6%)
		1 – 2 years	4	(7.7%)	0	(0%)	9	(10.5%)	11	(11%)	3	(25%)	3	(8.8%)	11	(17.2%)	6	(31.6%)
		More than 2 years	0	(0%)	0	(0%)	9	(10.5%)	2	(2%)	1	(8.3%)	7	(20.6%)	23	(35.9%)	0	(0%)
	Compensated (yes)		37	(71.2%)	3	(60%)	65	(75.6%)	49	(49%)	11	(91.7%)	17	(50%)	63	(98.4%)	18	(94.7%)
	Amount of Compensation	At 0%	15	(28.8%)	0	(0%)	21	(24.4%)	51	(51%)	1	(8.3%)	17	(50%)	1	(1.6%)	1	(5.3%)
		Less than 50%	0	(0%)	0	(0%)	3	(3.5%)	0	(0%)	0	(0%)	0	(0%)	6	(9.4%)	0	(0%)
		Between 50 and 74%	8	(15.4%)	1	(20%)	5	(5.8%)	19	(19%)	1	(8.3%)	1	(2.9%)	6	(9.4%)	0	(0%)
		Between 75and 99%	0	(0%)	1	(20%)	40	(46.5%)	20	(20%)	9	(75%)	10	(29.4%)	51	(79.7%)	17	(89.5%)
		Full or at 100%	29	(55.8%)	0	(0%)	17	(19.8%)	10	(10%)	1	(8.3%)	6	(17.6%)	0	(0%)	1	(5.3%)
Clinician's	Participating Clinicians		4		2		22		14		5		4		1		2	
	Participating Facilities		2		1		3		6		1		3		1		1	
	Age (years)		42.8	± 12.1	37.5	± 14.8	39.0	± 9.3	45.1	± 8.8	38.8	± 7.4	35	± 8.9	68		37	± 17.0
	Sex (female)		3	(75%)	1	(50%)	14	(63.6%)	5	(35.7%)	3	(60%)	3	(75%)	0	(0%)	1	(50%)
	Profession	Physical Therapist	3	(75%)	0	(0%)	20	(90.9%)	11	(78.6%)	3	(60%)	0	(0%)	1	(100%)	0	(0%)
		Occupational Therapist	1	(25%)	1	(50%)	2	(9.1%)	0	(0%)	2	(40%)	4	(100%)	0	(0%)	2	(100%)
		Other	0	(0%)	1	(50%)	0	(0%)	3	(21.4%)	0	(0%)	0	(0%)	0	(0%)	0	(0%)
	Experience	Years as Professional	22	(8.5-31.8)	15	(4-.)	14.5	(6-20)	17	(9.8-20.3)	10.5	(9.8-21.5)	11	(4.3-20.8)	45		13.8	(3.5-.)
		Years as FCE Assessor	3	(2.3-3)	13	(4-.)	5.5	(2.9-10.3)	8	(4.5-10.5)	8.5	(7.5-11.5)	6.5	(1.3-14.8)	19		7.3	(1.5-.)
		Nº FCE in the Last 2 Years [a]	52	(26.3-113.8)	10		24.5	(15-50)	90	(30-105)	15	(13.5-25)	214.5	(137.3-285)	200		775	(750-.)
	Pain Beliefs (Adapted BBQ) (9-45) [a]		38.5	(35.3-41.8)	45		42	(39.5-44)	39.5	(35.8-43)	37	/28.5-40)	35.5	(34.3-36.8)	41		41	(40-.)
FCE	Purpose	Admission for Rehabilitation	22	(42.3%)	0	(0%)	0	(0%)	100	(100%)	0	(0%)	0	(0%)	0	(0%)	9	(47.4%)
		(Pre-) employment Assessment	2	(3.8%)	0	(0%)	0	(0%)	0	(0%)	0	(0%)	0	(0%)	0	(0%)	0	(0%)
		Return-to-Work	22	(42.3%)	3	(60%)	67	(77.9%)	0	(0%)	12	(100%)	7	(20.6%)	39	(60.9%)	9	(47.4%)
		Case Settlement	3	(5.8%)	0	(0%)	7	(8.1%)	0	(0%)	0	(0%)	19	(55.9%)	3	(4.7%)	0	(0%)
		Determine Disability	3	(5.8%)	2	(40%)	12	(14.0%)	0	(0%)	0	(0%)	8	(23.5%)	22	(34.4%)	0	(0%)
		Other	0	(0%)	0	(0%)	0	(0%)	0	(0%)	0	(0%)	0	(0%)	0	(0%)	1	(5.3%)
	Direct Influence on Financial Status (yes)		0	(0%)	4	(80%)	66	(76.7%)	0	(0%)	12	(100%)	34	(100%)	63	(98.4%)	0	(0%)
	Type of Protocol	WorkWell	52	(100%)	0	(0%)	86	(100.0%)	100	(100%)	12	(100%)	34	(100%)	0	(0%)	0	(0%)
		WEST-EPIC	0	0 (0%)	5	(100%)	0	(0%)	0	(0%)	0	(0%)	0	(0%)	0	(0%)	19	(100%)
		Blankenship	0	0 (0%)	0	(0%)	0	(0%)	0	(0%)	0	(0%)	0	(0%)	64	(100%)	0	(0%)
	Floor-to-Waist Lift Performance																	
	Ending Reason	Normal End of Test	12	(23.1%)	1	(20%)	1	(1.2%)	54	(54%)	0	(0%)	25	(73.5%)	34	(53.1%)	19	(100%)
		Maximal Allowed HR was Reached	10	(19.2%)	0	(0%)	0	(0%)	0	(0%)	0	(0%)	0	(0%)	0	(0%)	0	(0%)
		Evaluator's Decision: Safety	7	(13.5%)	0	(0%)	5	(5.8%)	10	(10%)	0	(0%)	0	(0%)	0	(0%)	0	(0%)
		Evaluator's Decision: Max. Capacity	5	(9.6%)	2	(40%)	57	(66.3%)	5	(5%)	12	(100%)	1	(2.9%)	1	(1.6%)	0	(0%)
		Patient's Decision	16	(30.8%)	2	(40%)	23	(26.7%)	18	(18%)	0	(0%)	7	(20.6%)	29	(45.3%)	0	(0%)

		NL	CA	CH	DE	AT	ZA	NZ	CN
	Other: Maximum time exceeded	2 (3.8%)	0 (0%)	0 (0%)	0 (0%)	0 (0%)	0 (0%)	0 (0%)	0 (0%)
Six Minute Walk Test Performance									
Ending Reason	Normal End of Test	39 (75.0%)	5 (100%)	58 (67.4%)	11 (11%)	12 (100%)	32 (94.1%)	58 (90.6%)	19 (100%)
	Maximal Allowed HR was Reached	0 (0%)	0 (0%)	0 (0%)	0 (0%)	0 (0%)	0 (0%)	0 (0%)	0 (0%)
	Evaluator's Decision: Safety	0 (0%)	0 (0%)	0 (0%)	0 (0%)	0 (0%)	0 (0%)	0 (0%)	0 (0%)
	Evaluator's Decision: Max. Capacity	0 (0%)	0 (0%)	20 (23.3%)	1 (1%)	0 (0%)	0 (0%)	0 (0%)	0 (0%)
	Patient's Decision	0 (0%)	0 (0%)	8 (9.3%)	0 (0%)	0 (0%)	2 (5.9%)	6 (9.4%)	0 (0%)

Abbreviations: NL, The Netherlands; CA, Canada; CH, Switzerland; DE, Germany; AT, Austria; ZA, South Africa; NZ, New Zealand; CN, China; BMI, Body Mass Index; HR, Heart-Rate; DOT, Dictionary of Occupational Titles; PDI, Pain Disability Index; NRS, Numeric Rating Scale; WAS, Work Ability Score; FCE, Functional Capacity Evaluation; BBQ, Back Beliefs Questionnaire.

[a] median (IQR 25-75) values are given.

[b] Cultural Background measured as Nationality.

[c] Switzerland and South Africa have more than one official language: Switzerland = German (n=69), Italian (n=4), French (n=3); South Africa = Afrikaans (n=9), English (n=8), Sepedi (n=1), SeSotho (n=7), IsiZulu (n=7), Swazi (n=1), Tswana (n=1).

Other in Patient's Affected Body Area: South Africa = Thoracic Spine (n=1); New Zealand = Pelvic Floor Paralysis ex childbirth (n=1).

Other in Patient's Cultural Background: The Netherlands = Turkish (n=1); Canada = Chinese (n=1); Switzerland = Austrian (n=4), Congolese (n=1), German (n=7), Italian (n=4), Kosovar (n=2), Liechtensteiner (n=4), Macedonian (n=1), Portuguese (n=1), Serbian (n=1), Spanish (n=1), Turkish (n=1); Germany = Albanian (n=1), Italian (n=2), Polish (n=1), Russian (n=1), Serbian (n=1), Turkish (n=2); Austria= Croatian (n=1), German (n=1), Romanian (n=1); New Zealand = American (n=1), Chinese (n=1), Fijian-Indian (n=1), Indian (n=3), Iraqi (n=1), Tongan (n=2).

Other in Patient's Mother Language: The Netherlands = Ukrainian (n=1); Canada = Chinese (n=1); Switzerland = Albanian (n=5), Croatian (n=1), Serbian (n=1), Tamil (n=1), Turkish (n=2); Germany = Albanian (n=1), Panjabi (n=1), Polish (n=1), Serbian (n=1), Turkish (n=2); Austria = Bosnian (n=1), Croatian (n=1), Romanian (n=1), Serbian (n=1); New Zealand = Chinese (n=1), Tongan (n=2).

Other in Patient's Work Status: Switzerland = Student (n=1); New Zealand = Student (n=1), Voluntary job (n=1).

Other in FCE Purpose: China = Review work capacity for job duties suggestion (n=1).

Other in Clinician's Profession: Canada = Kinesiologist (n=1); Germany = Sports Scientist (n=1), Sports Teacher (n=2).
