# Supplementary material for: Functional Capacity Evaluation in Different Societal Contexts: Results of a Multicountry Study
Source: J Occup Rehabil. 2018 May 25;29(1):222–36. doi: 10.1007/s10926-018-9782-x (PMC6510856; doi:10.1007/s10926-018-9782-x)
Supplement: Supplementary file 3 — Supplementary material 3 (PDF 198 KB) [file 10926_2018_9782_MOESM3_ESM.pdf]

ELECTRONIC SUPPLEMENTARY MATERIAL

Title: Functional Capacity Evaluation in different societal contexts: Results of a multicountry study.

Journal: Journal of occupational rehabilitation.

Authors: Jone Ansuategui Echeita, Matthias Bethge, Berry J. van Holland, Douglas P. Gross, Jan Kool, Peter Oesch, Maurizio A. Trippolini, Elizabeth Chapman, Andy S.K. Cheng, Robert Sellars, Megan Spavins, Marco Streibelt, Peter van der Wurff, Michiel F. Reneman.

Corresponding Author: Jone Ansuategui Echeita, University of Groningen, University Medical Center Groningen, Department of Rehabilitation Medicine, Groningen, The Netherlands. Email: j.ansuategui.echeita@umcg.nl

Online Resource 3 Results from simple multilevel regression analyses with FCE test performances as dependent variables are given; each biopsychosocial factor's explained variance (R^2) are shown.

		Floor-to-Waist Lift [a]	Six Minute Walk [b]	Right Handgrip Strength [c]	Left Handgrip Strength [c]
**Bio**					
Patient's	Age (years)	0.02*	0.09***	0.03***	0.01**
	Sex (female=1)	0.15***	0.02	0.29***	0.33***
	Height (cm)	0.21***	0.10***	0.27***	0.30***
	Weight (kg)	0.09***	-0.02	0.11***	0.14***
	BMI (kg/m^2)	0.01	0.00**	0.00	0.01
	Affected Body Area	0.01**	0.11***	0.05***	0.05***
	Observed Physical Effort during Lift Test	0.17***	N.A.	N.A.	N.A.
	Test Ended Prematurely (yes=1)	0.09***	0.17***	N.A.	N.A.
	Pre-Test HR (bpm)	0.00	-0.01*	N.A.	N.A.
	Post-Test HR (bpm)	0.08***	0.10***	N.A.	N.A.
**Psycho**					
Patient-Reported	Pain Intensity (NRS)	0.19***	0.15***	0.07***	0.08***
	Pain Duration	0.04**	0.06***	0.00	0.00
	Effort during FCE Test (Borg CR-10)	0.01***	0.26***	N.A.	N.A.
	Anxiety	0.14***	0.07**	0.04**	0.03
	Social Isolation	0.17***	0.06**	0.05**	0.03*
	Catastrophizing	0.18***	0.10***	0.07***	0.06**
	Depression	0.12***	0.08***	0.02*	0.02
	Fear of Movement	0.09***	0.09***	0.01	0.01
	Disability (PDI)	0.18***	0.14***	0.04***	0.04**
	Work Ability - Single Item (WAS)	0.08***	0.05***	0.00*	0.01**
**Social**					
Patient's	Cultural Background [d]	0.10*	-0.26	0.11*	0.16*
	Mother Language	0.09*	-0.02	-0.02	-0.10
	Marital Status	-0.01	0.17	0.01	-0.01
	Educational Level	0.03*	0.07	0.01	0.03
	Physical Work Demands (DOT)	0.08***	0.00	0.16***	0.17***
	Days Off Work	0.07***	0.12***	0.00	-0.02
	Compensated (yes=1)	-0.01	0.00	0.00	0.01
Clinician's	Age (years)	0.00	-0.01	-0.04	-0.06
	Sex (female=1)	0.00	0.00	-0.01	-0.02
	Profession	0.00	-0.02	0.02	0.00
	Experience as Professional (years)	0.00	-0.01	-0.01	-0.02
	Experience as FCE Assessor (years)	-0.01	-0.01	-0.02	-0.02

	Nº FCE in the Last 2 Years	0.07*	0.11**	0.02	-0.02
	Pain Beliefs (Adapted BBQ)	0.00	-0.01	-0.01	-0.01
FCE	Purpose	-0.02	0.03	-0.04	-0.05
	Direct Influence in Financial Status (yes=1)	-0.01	-0.08	0.01	0.00
	Type of Protocol	0.05	N.A.	N.A.	N.A.

Abbreviations: BMI, Body Mass Index; HR, Heart-Rate; NRS, Numeric Rating Scale; PDI, Pain Disability Index; WAS, Work Ability Score; DOT, Dictionary of Occupational Titles; FCE, Functional Capacity Evaluation; BBQ, Back Beliefs Questionnaire; N.A., Not Applicable.

Significance: * < 0.05; ** < 0.01; *** < 0.001.

[a] measured in kg; [b] measured in m; [c] measured in kgF.

[d] Cultural Background measured as Nationality.

Reference category: Patient's Affected Body Area: Low Back; Observed Physical Effort by Clinicians: Light to Moderate; Patient's Pain Duration: Less than 6 months; Patient's Cultural Background: Dutch; Patient's Mother Language: English; Patient's Marital Status: Single; Patient's Educational Level: No Degree; Patient's Physical Work Demands: Sedentary; Patient's Days Off Work: No Days Off; Clinician's Profession: PT; FCE Purpose: Admission in a Rehabilitation Program; FCE Type of Protocol: WorkWell.
